# Toward a New Era in the Management of Hepatocellular Carcinoma: Novel Perspectives on Therapeutic Options and Biomarkers

**DOI:** 10.3390/ijms24109018

**Published:** 2023-05-19

**Authors:** Daniela Gabbia, Sara De Martin

**Affiliations:** Department of Pharmaceutical and Pharmacological Sciences, University of Padova, 35131 Padova, Italy

Liver cancer remains a global health challenge and its incidence is growing worldwide. The impact of hepatocellular carcinoma (HCC), the most common primary liver cancer, on global health is striking. In 2020, HCC was the sixth most diagnosed cancer, the fourth leading cause of cancer-related death, and the second leading cause of cancer-related mortality in men worldwide [1]. With 905,677 new cases and 830,180 new HCC-related deaths according to GLOBOCAN, this liver tumor has a high fatality rate, with incidence and mortality being quite comparable. Despite the improvements in the pharmacological treatment of locally advanced and advanced HCC, its prognosis remains poor, and the overall survival of HCC patients remains short [2].

In this scenario, many efforts have been made and are currently directed toward the identification of novel biomarkers and/or pharmacological targets to be exploited for the rational design of new drugs, as well as for the evaluation of combination therapy strategies. This Special Issue aimed at providing an overview of the variety of novel molecular targets, therapeutic options, and biomarkers for early diagnosis and/or as prognostic indicators of treatment responses that could be useful in drug development.

Many recent studies have been focused on finding biomarkers. The correlation between the tumor mutational burden (TMB), i.e., the total number of somatic non-synonymous mutations per coding area, and HCC prognosis has been investigated by many studies, as was reviewed by Gabbia and De Martin [3]. In general, there is a consensus that high TMB is associated with reduced survival, but it is also associated with an improved response to immunotherapy. However, its reliability as a biomarker that is useful for clinical decisions is far from being demonstrated. Likely, TMB needs to be integrated with other markers and specific mutation patterns to be effectively used as a decision tool for choosing the best pharmacological treatment.

A large cohort study investigated the potential association of two minor variants of sorting and assembly machinery component 50 homolog (SAMM50) with HCC in patients with alcohol-associated liver disease [4]. The presence of SAMM50 rs3827385 and rs3761472 risk alleles has been linked to the risk factor PNPLA3 148M, whose presence increases the risk of developing alcoholic-related HCC in wildtype SAMM50.

Recently, much attention has been paid to the diagnostic potential of liquid biopsies. The study by Manganelli and collaborators investigated, for the first time, the expression of telomeric repeat-containing RNA (TERRA) in HCC [5]. Since TERRA localized at telomeres interacts with the telomerase RNA subunit (TERC) and with the telomerase catalytic subunit (TERT), this study determined TERRA, TERT, and TERC in tumoral and peritumoral tissues and in plasma obtained from healthy and HCC donors. This study demonstrates that TERRA were downregulated and the TERT upregulated in HCC tissue with respect to the peritumoral specimens. Circulating TERRA and TERC levels were higher in HCC patients than in healthy donors. Moreover, HCC cells are able to secrete TERRA and TERC transcripts into extracellular vesicles and sorafenib increased their release. Thus, this study presents a new perspective on the role of TERRA in HCC cancer cell biology and suggests that irrespective of the origin of circulating TERRA, plasma TERRA and TERC levels deserve recognition as non-invasive tools for HCC diagnosis.

Many efforts have been devoted to unraveling the pathological mechanisms underlining the progression of chronic liver diseases to HCC, with the final aim being to find druggable targets to prevent or cure HCC. In this regard, the study of Andrade et al. suggests that the inhibition of the group VIA phospholipase A2 (iPLA2β), a protein involved in the regulation of epithelial cells and macrophage functions, could prevent HCC by inducing cell-cycle arrest [6]. They demonstrated that iPLA2β KO mice have an improved resistance to diethylnitrosamine (DEN)-induced HCC that is likely to reside in the suppression of the hepatic expression of cyclin D2 (CCND2) and B-cell lymphoma 2 (BCL2), which are involved in cell-cycle regulation, and of IL6, IL10, and VCAM-1, which are involved in inflammatory processes. iPLA2β deficiency in macrophages has been associated with ferroptosis, which may affect mitosis and hepatic cell regeneration that prevent nodular formation and HCC development.

For over a decade, the TKI inhibitor sorafenib has represented the main therapeutic option for advanced HCC. In the last few years, new therapeutic regimens have been approved as first- and/or second-line treatments, including immunotherapeutic agents and combinations, which have also shown promising results. A study performed on a rat model of DEN-induced HCC investigated the effect on tumor growth of the next-generation AKT inhibitor vevorisertib, which is characterized by improved pharmacokinetic and pharmacodynamic features compared to miransertib, another allosteric inhibitor currently under investigation for HCC treatment [7]. This study demonstrated that vevorisertib, alone or in combination with sorafenib, was able to significantly reduce tumor proliferation, normalize hepatic vasculature, and improve liver fibrosis, suggesting that targeting the AKT pathway maybe a valuable therapeutic option for an HCC cure.

The study of Okumura and colleagues reported a positive role of the hepatocyte growth factor (HGF) in increasing the efficacy of chemotherapy with fluoropyrimidine drugs, e.g., 5-fluorouracil (5-FU), suggesting that the HGF/cMet axis plays a crucial role in the safety and success of this chemotherapy regimen [8]. This study also reported that the tyrosine kinase inhibitor erlotinib can regulate the HGF/cMet axis by enhancing the expression of UPP1, a protein involved in 5-FU metabolism. It is also able to regulate the HGF-related increased phosphorylation of extracellular signal-regulated kinases (ERKs), synergistically leading to an increased anticancer effect.

In their review, Scagliola and collaborators summarized the relevant findings about the role of the eukaryotic initiation factors (eIFs) that control the hepatic translational machinery regulating the mRNA transcription of the oncogenic signaling cascade during HCC development [9]. Since these factors have been recently proposed as valuable biomarkers and druggable targets, this review provides an overview of their feasible use in current systemic therapies and how they could represent an innovative target for HCC treatment.

In conclusion, HCC is likely to gain more attention in the next few years within the oncology scenario due to its high mortality and growing incidence. The efforts of the scientific community must be driven toward the optimization of our current knowledge and the search for new approaches in finding feasible biomarkers for early diagnosis and clinical decisions, as well as specific druggable targets for novel therapies.
